# Supplementary material for: Identification and Functional Characterization of General Odorant Binding Proteins in Orthaga achatina
Source: Insects. 2023 Feb 22;14(3):216. doi: 10.3390/insects14030216 (PMC10051560; doi:10.3390/insects14030216)
Supplement: Supplementary file 1 [file insects-14-00216-s001.zip › Figure S2.pdf]

OachGOBP2

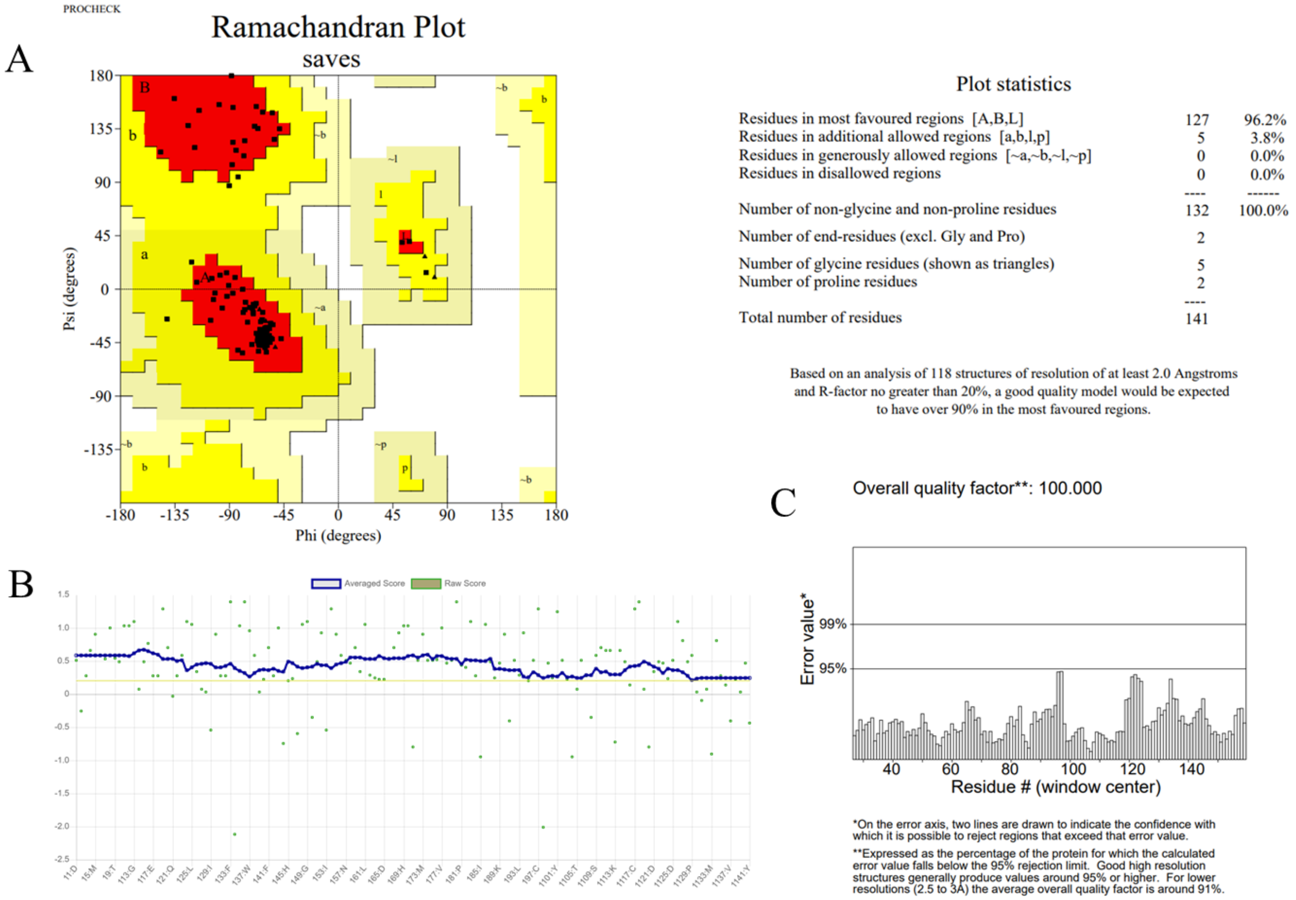

Figure S2. Evaluation of OachGOBP2. A. Ramachandran plot. B. Verify 3D graph. C. ERRAT generated result.
