# Supplementary material for: Identification and Functional Characterization of General Odorant Binding Proteins in Orthaga achatina
Source: Insects. 2023 Feb 22;14(3):216. doi: 10.3390/insects14030216 (PMC10051560; doi:10.3390/insects14030216)
Supplement: Supplementary file 1 [file insects-14-00216-s001.zip › Table S1.pdf]

Table S1. Primers used in this study.

Primer name	Sequence（5'-3'）	
	**Forward primer**	**Reverse primer**
**Primers for RT-PCR**		
GOBP1-F/R	GCAGACGTTGTGGTTATGAAA	CTAAGCTTCTGCTTCCATAATGA
GOBP2-F/R	GATGCTGAAGTGATGAGCCA	CTAATATTTCTCCATGACGGCT
β-Actin-F/R	GAGAGGGAAATCGTTCGTGACATC	GGAAGCGGCAGCGGTAGC
**Primers for qPCR**		
GOBP1-F/R	CGTTGTGGTTATGAAAGATG	CGGTGAGAAGGTTGAAG
GOBP2-F/R	TCTCGTTGGTGCTACT	CTTGGCGACACTCTTC
β-Actin-F/R	CTCTGGTGATGGTGTCTC	GAGGTAGTCGGTCAAGTC
GAPDH-F/R	GCTGAGTATGTTGTAGAATCC	GGAGCAGAGATGATAACCT
**Primers for Prokaryotic expression**		
GOBP1-F/R	GCCATGGCTGATATCGGATCCGCAGACGTTGTGGTTATGAA	GTGGTGGTGGTGGTGCTCGAGCTAAGCTTCTGCTTCCATAATG
GOBP2-F/R	GCCATGGCTGATATCGGATCCGATGCTGAAGTGATGAGCCA	GTGGTGGTGGTGGTGCTCGAGCTAATATTTCTCCATGAC
