# Supplementary material for: Identification and Functional Characterization of General Odorant Binding Proteins in Orthaga achatina
Source: Insects. 2023 Feb 22;14(3):216. doi: 10.3390/insects14030216 (PMC10051560; doi:10.3390/insects14030216)
Supplement: Supplementary file 1 [file insects-14-00216-s001.zip › Table S2.pdf]

**Table S2.** Accession numbers for GOBPs used in the phylogenetic analysis.

| Species | Gene Name | Acc. number |
|---|---|---|
| *Amyelois transitella* | AtraGOBP1 | ACX47893.1 |
| | AtraGOBP2 | ACX47894.1 |
| *Antheraea pernyi* | AperGOBP1 | CAA71866.1 |
| | AperGOBP2 | CAA65575.1 |
| *Bombyx mori* | BmorGOBP1 | NP_001037496.1 |
| | BmorGOBP2 | NP_001037498.1 |
| *Chilo suppressalis* | CsupGOBP1 | ACJ07129.1 |
| | CsupGOBP2 | ACJ07120.1 |
| *Ectropis obliqua* | EoblGOBP1 | ACN29680.1 |
| | EoblGOBP2 | ACN29681.1 |
| *Epiphyas postvittana* | EposGOBP1 | Q95VP3.1 |
| | EposGOBP2 | AAL05869.1 |
| *Grapholita molesta* | GmolGOBP1 | AFH02841.1 |
| | GmolGOBP2 | AFH02842.1 |
| *Helicoverpa armigera* | HarmGOBP1 | XP_021192665.1 |
| | HarmGOBP2 | CAC08211.1 |
| *Helicoverpa assulta* | HassGOBP1 | AAW65076.1 |
| | HassGOBP2 | AAQ54909.1 |
| *Mythimna separata* | MsepGOBP1 | AWT22240.1 |
| | MsepGOBP2 | AWT22242.1 |
| *Operophtera brumata* | ObruGOBP1 | KOB67878.1 |
| | ObruGOBP2 | KOB70852.1 |
| *Ostrinia nubilalis* | OnubGOBP1 | BBB15959.1 |
| | OnubGOBP2 | BBB15969.1 |
| *Spodoptera exigua* | SexiGOBP1 | ACY78412.1 |
| | SexiGOBP2 | CAC12832.1 |
| *Spodoptera litura* | SlitGOBP1 | ABM54823.1 |
| | SlitGOBP2 | ABM54824.1 |
| *Streltzoviella insularis* | SinsGOBP1 | QLI62030.1 |
| | SinsGOBP2 | QLI62031.1 |
