# Supplementary material for: Identification and Functional Characterization of General Odorant Binding Proteins in Orthaga achatina
Source: Insects. 2023 Feb 22;14(3):216. doi: 10.3390/insects14030216 (PMC10051560; doi:10.3390/insects14030216)
Supplement: Supplementary file 1 [file insects-14-00216-s001.zip › Table S3.pdf]

Table S3. Sex pheromones and plant volatiles used in this study.

Ligands		Purity	CAS NO.
**Sex pheromone**			
(Z11)-Hexadecenol	Z11-16: OH	>95%	56683-54-6
(Z11)-Hexadecenyl acetate	Z11-16: OAc	>95%	34010-21-4
(Z11)-Hexadecenal	Z11-16: Ald	>95%	53939-28-9
(3Z,6Z,9Z,12Z,15Z)-3,6,9,12,15- Tricosapentaene	Z3,Z6,Z9,Z12,Z15-23: H	>95%	854201-94-8
**Plant volatiles**			
1-Hexanol		98%	111-27-3
(Z3)-Hexenyl acetate		98%	3681-71-8
(Z3)-Hexenal		98%	6789-80-6
(Z3)-Hexen-1-ol		98%	928-96-1
β-Pinene		>95%	19902-08-0
Hexyl acetate		99%	142-92-7
E2-Hexenal		98%	6728-26-3
β-Ionone		96%	14901-07-6
α-Ionone		90%	127-41-3
Farnesol		95%	4602-84-0
α-Farnesene		>95%	502-61-4
α-Phellandrene		>95%	99-83-2
Linalool		95%	78-70-6
Ocimene		90%	13877-91-3
Cinnamaldehyde		98%	104-55-2
Camphor		>95%	76-22-2
Eucalyptol		>95%	470-82-6
Valeraldehyde		>95%	110-62-3
Geraniol		98%	106-24-1
Limonene		99%	5989-27-5
β-Farnesene		98%	18794-84-8
Camphene		>95%	79-92-5
