# Supplementary material for: Identification and Functional Characterization of General Odorant Binding Proteins in Orthaga achatina
Source: Insects. 2023 Feb 22;14(3):216. doi: 10.3390/insects14030216 (PMC10051560; doi:10.3390/insects14030216)
Supplement: Supplementary file 1 [file insects-14-00216-s001.zip › Table S4.pdf]

Table S4. Prediction of key amino acid residues during the docking of OachGOBPs to different ligands.

Ligand	Different residues within 4.0 Å[a]		Residues forming H-bond with ligand	
	GOBP1	GOBP2	GOBP1	GOBP2
**Plant volatiles**				
Z3-Hexenyl acetate	-	M27, V30, T31, L83, Y98, R132	-	T31(O···H-O=2.8Å)
Z3-Hexen-1-ol	-	M27, V30, T31,T88, I90, L83, R89,R132	-	T88(O-H···O=2.6Å) R132(O···H-NH=2.2Å)
β-Ionone	-	M27, V30, T31, T88, I90, L83, Y98, R89,R132	-	S78(O-H···O=2.9Å)
Farnesol	S89, R90	M27, V30, T31, T88, I90, L83, Y98, R89,R132	T32(O-H···O=2.1Å) W60(O···H-NH=2.3Å)	N
α-Farnesene	-	M27, V30, T31, I90, L83, Y98, R132	-	N
α-Phellandrene	-	M27, V30, T31, T88, I90, L83, Y98, R132	-	N
Linalool	-	M27, V30, T31, I90, L83, Y98, R132	-	T31(O-H···O=2.0Å) W59(O···H-NH=2.3Å)
Camphor	-	I90	-	N
**Sex pheromone**				
Z11-16: OH	Common	M27, V30, T31, T88, I90, L83, Y98, R89,R132	W60(O···H-NH=2.5Å)	N
Z11-16: OAc	-	M27, V30, T31, T88, I90, L83, Y98, R89,R132	-	S78(O···H-NH=3.0Å, O···H-O=2.8Å)

Note: "N" means no hydrogen bond formation of OachGOBPs and ligands.

(X···Y=ZÅ), X represents the atoms on the ligand, ··· means the hydrogen bond, Y represents the atom on the amino acid residue, and Z represents the distance of hydrogen bond between OachGOBPs and ligands. A shorter distance indicates a stronger interaction between OachGOBPs and ligands.

[a] Common amino acid residues in the binding pockets of OachGOBP1 or 2 with 4.0 Å are shown as follows: GOBP1: M28, M91, M113, V31, V137, T32, T96, F35, F56, F59, F99, F141, W60, I75, I117, I134, S79, L84, L85, H92, E121, R133 and A138; GOBP2: M95, M112, V133, V136, F34, F55, F58, F140, W59, I74, I116, S78, L84, E120 and A137.
