# Supplementary material for: Identification and Functional Characterization of General Odorant Binding Proteins in Orthaga achatina
Source: Insects. 2023 Feb 22;14(3):216. doi: 10.3390/insects14030216 (PMC10051560; doi:10.3390/insects14030216)
Supplement: Supplementary file 1 [file insects-14-00216-s001.zip › Tables S5-S11.pdf]

Table S5. The distance between the partial carbon atoms on the amino acid residues of OachGOBP1 and each farnesol atom.

OachGOBP1 / Farnesol atom	THR32-CA	THR32-CB	THR32-CG2	TRP60-CD1	TRP60-CE2	TRP60-CZ2	Structure
**C1**	5.1 Å	4.7 Å	4.8 Å	5.0 Å	4.1 Å	3.8 Å	
**C2**	6.0 Å	5.4 Å	5.7 Å	6.1 Å	5.3 Å	5.0 Å	
**C3**	6.4 Å	6.4 Å	6.4 Å	7.3 Å	6.6 Å	6.2 Å	
**C4**	5.8 Å	6.2 Å	6.4 Å	7.9 Å	7.1 Å	6.7 Å	
**C5**	7.6 Å	7.8 Å	7.6 Å	8.5 Å	7.8 Å	7.5 Å	
**C6**	8.8 Å	8.7 Å	8.5 Å	8.7 Å	7.9 Å	7.5 Å	
**C7**	10.0 Å	10.0 Å	9.8 Å	10.1 Å	9.2 Å	8.6 Å	
**C8**	10.4 Å	10.5 Å	10.5 Å	10.6 Å	9.4 Å	8.6 Å	
**C9**	9.8 Å	9.8 Å	10.0 Å	9.9 Å	8.4 Å	7.5 Å	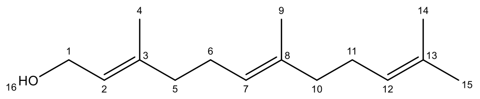
**C10**	11.7 Å	11.9 Å	11.9 Å	12.1 Å	10.9 Å	10.0 Å	
**C11**	11.8 Å	12.2 Å	12.3 Å	12.9 Å	11.8 Å	11.0 Å	
**C12**	11.5 Å	12.0 Å	12.3 Å	13.1 Å	11.8 Å	10.9 Å	
**C13**	10.5 Å	11.0 Å	11.4 Å	12.5 Å	11.3 Å	10.5 Å	
**C14**	9.3 Å	9.9 Å	10.2 Å	11.5 Å	10.5 Å	9.8 Å	
**C15**	10.5 Å	11.2 Å	11.7 Å	13.0 Å	11.7 Å	10.8 Å	
**O16**	4.4 Å	3.7 Å	3.5 Å	4.0 Å	3.7 Å	3.8 Å	

Note: "partial carbon atoms" refers to the carbon atom on an amino acid hydrogen-bonded to the ligand and within the range of ligand 4 Å.

The atoms on all ligands (except hydrogen atoms) are numbered for statistical purposes. The atom numbers are shown in the structure column.

Table S6. The distance between the partial carbon atoms on the amino acid residues of OachGOBP1 and each Z11-16: OH atom.

OachGOBP1 / Z11-16: OH atom	TRP60-CD1	TRP60-CE2	TRP60-CZ2	TRP60-CH2	Structure
**C1**	8.3 Å	7.6 Å	7.1 Å	8.1 Å	
**C2**	9.1 Å	8.2 Å	7.5 Å	8.3 Å	
**C3**	10.5 Å	9.6 Å	9 Å	9.7 Å	
**C4**	11.0 Å	10.1 Å	9.5 Å	10.1 Å	
**C5**	12.3 Å	11.2 Å	10.4 Å	10.9 Å	
**C6**	13.0 Å	11.8 Å	11.0 Å	11.3 Å	
**C7**	12.7 Å	11.7 Å	10.9 Å	11.2 Å	
**C8**	12.9 Å	11.7 Å	10.9 Å	10.9 Å	
**C9**	11.7 Å	10.4 Å	9.6 Å	9.6 Å	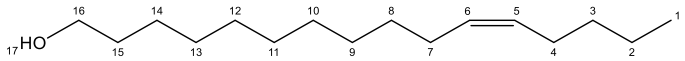
**C10**	10.9 Å	9.4 Å	8.5 Å	8.5 Å	
**C11**	9.6 Å	8.2 Å	7.4 Å	7.5 Å	
**C12**	8.5 Å	7.3 Å	6.6 Å	6.8 Å	
**C13**	8.4 Å	7.5 Å	7.1 Å	7.5 Å	
**C14**	7.2 Å	6.5 Å	6.2 Å	6.9 Å	
**C15**	6.1 Å	5.2 Å	4.8 Å	5.5 Å	
**C16**	4.8 Å	4.3 Å	4.2 Å	5.2 Å	
**O17**	4.5 Å	3.5 Å	3.2 Å	4.4 Å	

Table S7. The distance between the partial carbon atoms on the amino acid residues of OachGOBP2 and each Z3-Hexen-1-ol atom.

OachGOBP2 / Z3-Hexen-1-ol atom	THR88-C	ARG132-CZ	Structure
C1	10.3 Å	10.3 Å	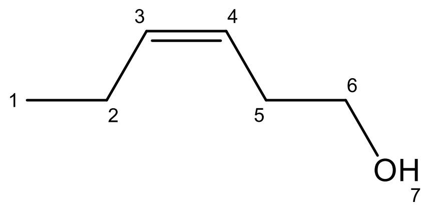
C2	8.8 Å	9.2 Å	
C3	8.3 Å	8.3 Å	
C4	7.1 Å	7.0 Å	
C5	6.0 Å	6.3 Å	
C6	5.3 Å	4.8 Å	
O7	3.9 Å	4.2 Å	

Table S8. The distance between the partial carbon atoms on the amino acid residues of OachGOBP2 and each Linalool atom.

OachGOBP2 / Linalool atom	THR31-CA	THR31-CB	THR31-CG2	TRP59-CE2	TRP59-CZ2	Structure
C1	4.8 Å	4.7 Å	4.8 Å	4.6 Å	4.3 Å	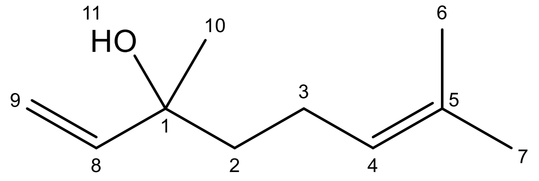
C2	4.5 Å	4.8 Å	4.9 Å	6.0 Å	5.8 Å	
C3	4.9 Å	5.6 Å	6.0 Å	6.9 Å	6.4 Å	
C4	6.2 Å	6.9 Å	7.2 Å	8.2 Å	7.7 Å	
C5	7.4 Å	8.1 Å	8.3 Å	8.6 Å	8.0 Å	
C6	7.8 Å	8.2 Å	8.5 Å	8.0 Å	7.2 Å	
C7	8.7 Å	9.4 Å	9.5 Å	10.0 Å	9.4 Å	
C8	6.2 Å	6.0 Å	5.8 Å	4.9 Å	4.6 Å	
C9	7.0 Å	6.9 Å	6.5 Å	6.1 Å	5.9 Å	
C10	5.1 Å	4.9 Å	5.4 Å	4.3 Å	3.7 Å	
O11	4.1 Å	3.7 Å	3.5 Å	3.7 Å	3.9 Å	

Table S9. The distance between the partial carbon atoms on the amino acid residues of OachGOBP2 and each β-Ionone atom.

OachGOBP2 / β-Ionone atom	SER78-C	Structure
**O1**	4.1 Å	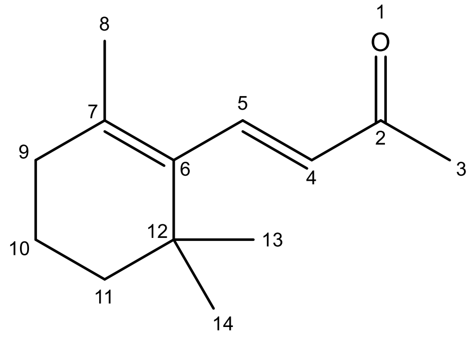
**C2**	5.0 Å	
**C3**	5.9 Å	
**C4**	5.7 Å	
**C5**	5.5 Å	
**C6**	6.5 Å	
**C7**	6.8 Å	
**C8**	6.3 Å	
**C9**	8.1 Å	
**C10**	8.9 Å	
**C11**	9.0 Å	
**C12**	7.7 Å	
**C13**	7.4 Å	
**C14**	8.3 Å	

Table S10. The distance between the partial carbon atoms on the amino acid residues of OachGOBP2 and each Z11-16: OAc atom.

OachGOBP2 / Z11-16: OAc atom	SER78-CB	Structure
**C1**	7.4 Å	
**C2**	7.7 Å	
**C3**	8.4 Å	
**C4**	8.2 Å	
**C5**	7.8 Å	
**C6**	8.2 Å	
**C7**	9.1 Å	
**C8**	9.5 Å	
**C9**	10.6 Å	
**C10**	10.3 Å	
**C11**	9.9 Å	
**C12**	8.5 Å	
**C13**	7.8 Å	
**C14**	6.3 Å	
**C15**	6.2 Å	
**C16**	5.8 Å	
**O17**	4.5 Å	
**C18**	4.6 Å	
**C19**	5.8 Å	
**O20**	4.1 Å	

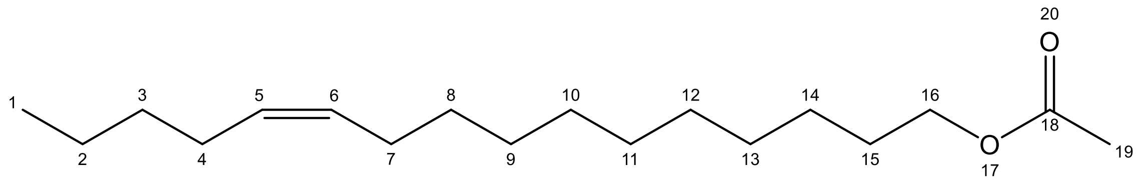

Table S11. The distance between the partial carbon atoms on the amino acid residues of OachGOBP2 and each Z3-Hexenyl acetate atom.

OachGOBP2 / Z3-Hexenyl acetate atom	THR31-CA	THR31-CB	THR31-CG2	Structure
**C1**	4.4 Å	5.1 Å	5.5 Å	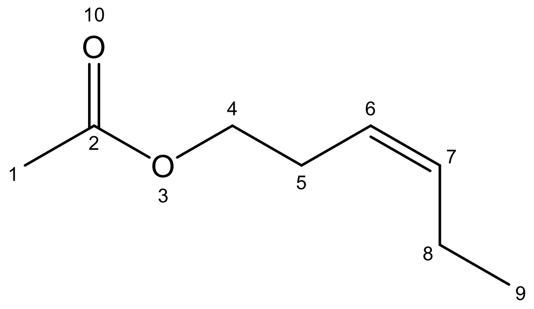
**C2**	3.7 Å	4.0 Å	4.5 Å	
**O3**	4.1 Å	4.1 Å	4.1 Å	
**C4**	5.3 Å	4.9 Å	4.9 Å	
**C5**	6.5 Å	6.3 Å	6.2 Å	
**C6**	6.8 Å	6.6 Å	6.2 Å	
**C7**	7.6 Å	7.7 Å	7.2 Å	
**C8**	8.4 Å	8.6 Å	8.3 Å	
**C9**	8.0 Å	8.5 Å	8.4 Å	
**O10**	3.5 Å	3.7 Å	4.6 Å	
