# Supplementary figures and images for: Identification and Functional Characterization of General Odorant Binding Proteins in Orthaga achatina
Source: Insects. 2023 Feb 22;14(3):216. doi: 10.3390/insects14030216 (PMC10051560; doi:10.3390/insects14030216)

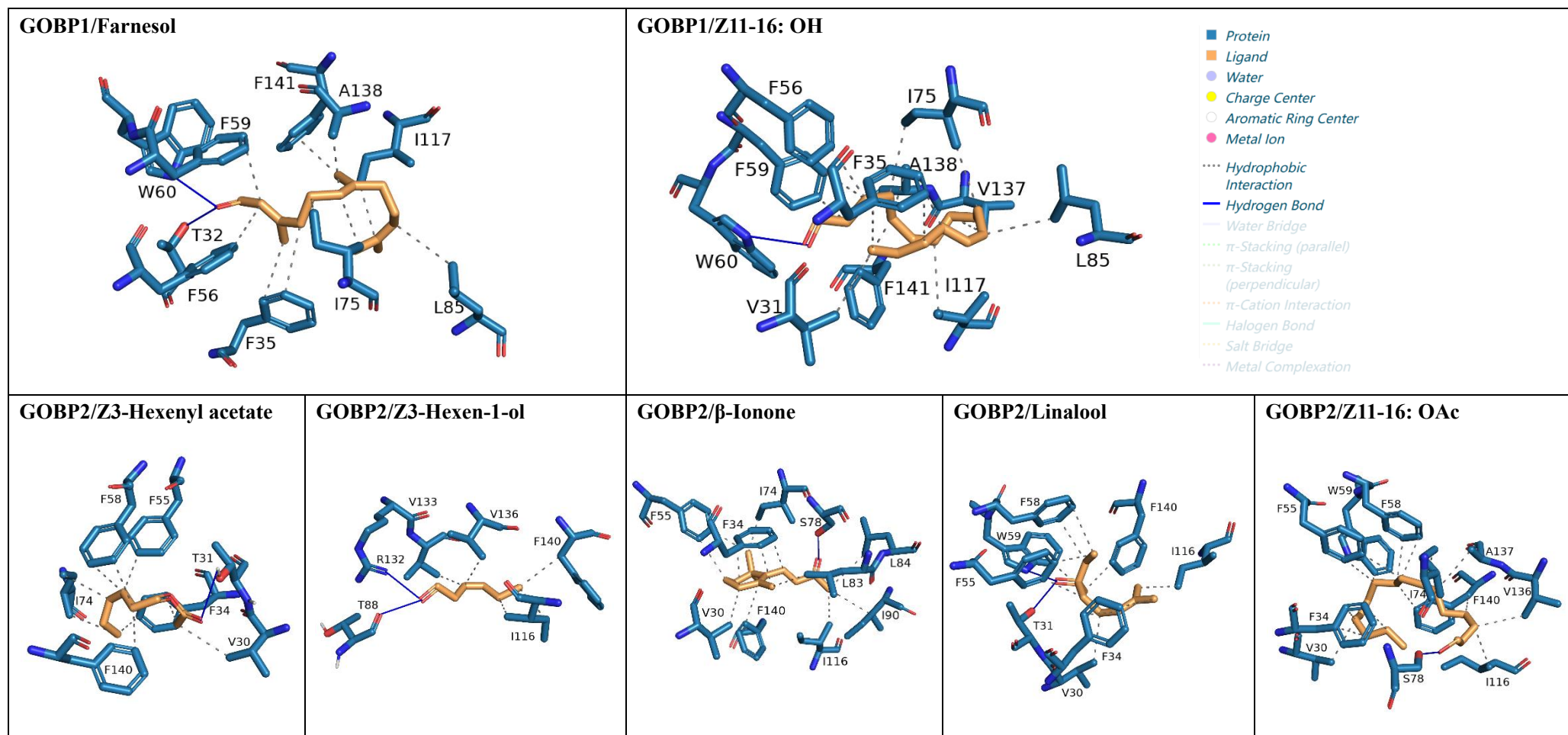

**Figure S3.** 2D-plots of contacts showing the GOBPs-ligands interactions.

Supplement: Supplementary file 1 [file insects-14-00216-s001.zip › Figure S3.pdf]
